# Dental Office and Laboratory

**Published:** 1877-04

**Authors:** 


### DENTAL OFFICE AND LABORATOY.

The publication of this sprightly little quarterly, has, after a suspension of four years, been again resumed. The first number of the new issue is at hand. Though largely directed to advertising, it always contains some good, practical matter. The present number has a good article on "Congestion of the Pulp," from the pen of Prof. G. T. Barker, which is worth many times the price of the paper for a year.

